# A national population-based study of cannabis use and correlates among U.S. veterans prescribed opioids in primary care

**DOI:** 10.1186/s12888-023-04648-5

**Published:** 2023-03-17

**Authors:** Tauheed Zaman, Dawn M. Bravata, Amy L. Byers, Erin E. Krebs, Samuel J. Leonard, Friedhelm Sandbrink, Wylie Barker, Salomeh Keyhani

**Affiliations:** 1 Addiction Recovery and Treatments Services, San Francisco VA Health Care System, San Francisco, CA USA; 2 Department of Psychiatry and Behavioral Sciences, University of California, San Francisco, CA USA; 3 Richard L. Roudebush VA Medical Center, Indianapolis, IN USA; 4 Departments of Medicine and Neurology, Indiana University School of Medicine, Indianapolis, IN USA; 5 Department of Medicine, University of California, San Francisco, CA USA; 6 San Francisco VA Medical Center, San Francisco, CA USA; 7 Center for Care Delivery and Outcomes Research, Minneapolis VA Health Care System, Minneapolis, MN USA; 8 Department of Medicine, University of Minnesota Medical School, Minneapolis, MN USA; 9 Northern California Institute for Research and Education, San Francisco, CA USA; 10 National Pain Management, Opioid Safety and Prescription Drug Monitoring Program, Veterans Health Administration, Washington, DC USA; 11 Department of Neurology, George Washington University, Washington, DC USA

**Keywords:** Cannabis, Opioid therapy, Opiates, Veterans

## Abstract

**Background:**

Cannabis is marketed as a treatment for pain. There is limited data on the prevalence of cannabis use and its correlates among Veterans prescribed opioids.

**Objective:**

To examine the prevalence and correlates of cannabis use among Veterans prescribed opioids.

**Design:**

Cross-sectional study.

**Participants:**

Veterans with a urine drug test (UDT) from Primary Care 2014–2018, in 50 states, Washington, D.C., and Puerto Rico. A total of 1,182,779 patients were identified with an opioid prescription within 90 days prior to UDT.

**Main measures:**

Annual prevalence of cannabis positive UDT by state. We used multivariable logistic regression to assess associations of demographic factors, mental health conditions, substance use disorders, and pain diagnoses with cannabis positive UDT.

**Results:**

Annual prevalence of cannabis positive UDT ranged from 8.5% to 9.7% during the study period, and in 2018 was 18.15% in Washington, D.C. and 10 states with legalized medical and recreational cannabis, 6.1% in Puerto Rico and 25 states with legalized medical cannabis, and 4.5% in non-legal states. Younger age, male sex, being unmarried, and marginal housing were associated with use (*p* < 0.001). Post-traumatic stress disorder (adjusted odds ratio [AOR] 1.17; 95% confidence interval [CI] 1.13–1.22, *p* < 0.001), opioid use disorder (AOR 1.14; CI 1.07–1.22, *p* < 0.001), alcohol use disorder or positive AUDIT-C (AOR 1.34; 95% CI 1.28–1.39, *p* < 0.001), smoking (AOR 2.58; 95% CI 2.49–2.66, *p* < 0.001), and other drug use disorders (AOR 1.15; 95% CI 1.03–1.29, *p* = 0.02) were associated with cannabis use. Positive UDT for amphetamines AOR 1.41; 95% CI 1.26–1.58, *p* < 0.001), benzodiazepines (AOR 1.41; 95% CI 1.31–1.51, *p* < 0.001) and cocaine (AOR 2.04; 95% CI 1.75–2.36, *p* < 0.001) were associated with cannabis positive UDT.

**Conclusions:**

Cannabis use among Veterans prescribed opioids varied by state and by legalization status. Veterans with PTSD and substance use disorders were more likely to have cannabis positive UDT. Opioid-prescribed Veterans using cannabis may benefit from screening for these conditions, referral to treatment, and attention to opioid safety.

**Supplementary Information:**

The online version contains supplementary material available at 10.1186/s12888-023-04648-5.

## Background

Cannabis use has increased over the past decade [1], with past-month cannabis use in the United States (U.S.) almost doubling from 5.8% in 2007 to 11.5% in 2019 [2]. The most recent National Survey of Drug Use and Health indicated the highest rates of past-month use among 18–25-year-olds (23%) compared to other age groups, as well as a steady rise among adults 26 years or older, with 7.9%, 8.6%, and 10.2% reporting past month use in 2017, 2018, and 2019, respectively [3, 4]. Cannabis is marketed as a treatment of pain [5] and there is some emerging evidence that it may be beneficial in the management of pain [6]. Therefore, use among patients prescribed opioid analgesic therapy for pain management may be common. Given the current and projected growth of cannabis use in the U.S., understanding the prevalence of cannabis use in those prescribed opioids has important implications for clinical care.

To date, there are no published national data that quantify the prevalence and correlates of cannabis use among U.S. adults prescribed opioids. Previous studies of chronic pain patients have indicated that depression, anxiety, opioid use disorder, tobacco, alcohol, cocaine, and sedative use [7, 8] are associated with cannabis use. Additionally, studies of chronic pain patients include mixed and low-quality evidence on the opioid-sparing properties of cannabis [9, 10]. A recent prospective cohort study suggested that cannabis use does not decrease opioid use among patients prescribed cannabis for non-cancer pain [11]. Cannabis use among adults prescribed opioid therapy is a concern due to potential harms associated with combined use of two psychoactive drugs.

Data from the Veterans’ Health Administration (VHA), the nation’s largest healthcare system, provides a unique opportunity to examine the prevalence of cannabis use and correlates of use among patients prescribed opioids in primary care. In 2014, VHA recommended routine annual urine drug testing (UDT) for all patients prescribed opioids for greater than 90 days in primary care and began tracking compliance with this metric at a national level [12–15]. This population-based study reports on the annual prevalence of cannabis positive UDT within the VHA during 2014–2018, state-level variation in prevalence according to the legal status of cannabis in 2018, and the association of Veteran demographic factors, mental health diagnoses, substance use disorders and pain diagnoses with cannabis positive UDT.

## Methods

### Sample: Opioid-prescribed veterans

Using data from the VHA Corporate Data Warehouse (CDW) [16], we identified Veterans aged 18 or older who received a UDT in primary care between 2014 and 2018 using a previously developed algorithm [17, 18]. All UDTs were completed at outpatient laboratories. The sample included Veterans in all 50 states, the District of Columbia, and Puerto Rico.

**Fig. 1:**
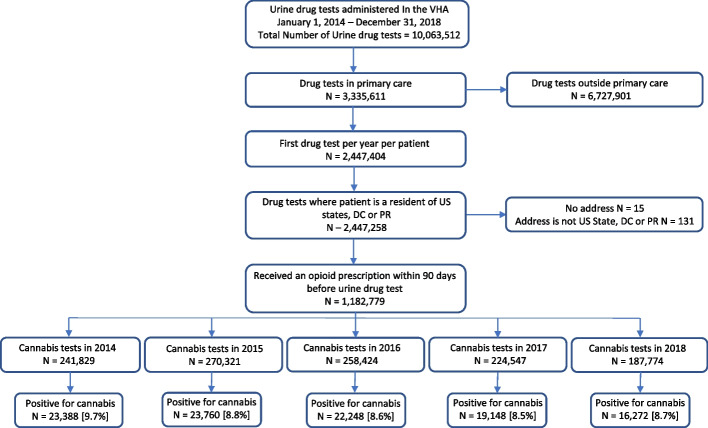
Data source and flow diagram to determine prevalence of cannabis use among all US Veterans 18 years and older prescribed opioids 2014–2018

We chose to evaluate screening in primary care clinics as most opioids are prescribed in this setting. This outpatient setting also allows for examination of data over longer periods of time in comparison to inpatient settings, and in an environment likely reflective of the patient’s usual patterns of use outside of a closely monitored acute care unit. As all Veterans who receive opioids for greater than 90 days are recommended to receive annual urine drug testing, there is less possibility of selection bias in who receives screening. We restricted the sample to the first UDT for each Veteran in primary care (*N* = 2,447,258) per year. We further restricted the sample to Veterans each year who had filled at least one prescription for an opioid analgesic medication in the 90 days prior to the UDT (*N* = 1,182,779) (Fig. 1).

### Data sources and measurements

The primary dependent variable of interest was the presence of cannabis in the UDT. These tests detect the presence of tetrahydrocannabinol (THC) and can remain positive for up to 30 days after use [19], and we considered a positive result a reasonable proxy for cannabis use. Independent variables included sociodemographic factors, non-substance use mental health diagnoses, substance use disorders, and pain diagnoses. We included these variables as other studies have demonstrated the association of these domains with cannabis use [20, 21]. Pain diagnoses were examined as these were the indications for opioid prescription, and because cannabis is frequently marketed as a treatment for a range of pain syndromes [22].

To identify non-substance use mental health diagnoses, we used the presence of two International Classification of Disease-9 (ICD-9) and ICD-10 outpatient codes in the past two years, or the presence of one inpatient code. We used all data sources available including national VA data, Medicare data and VA community care files to identify ICD codes. We searched for codes for common mental health diagnoses: psychosis, depression, bipolar disorder, PTSD, and anxiety (Table 1). We used a similar approach to identify chronic pain, and searched for codes indicating common pain diagnoses: back pain, neck pain, osteoarthritis, neuropathy, and headache.

**Table 1:** Baseline characteristics of veterans 18 years and older on prescription opioid therapy who were screened for cannabis via urine toxicology (2018)

N	All	Negative cannabis	Positive cannabis	*P*-Value
	187,774	171,502	16,272	
**Age**				
< 55	36,800 (19.6)	33,040 (19.3)	3760 (23.1)	< 0.001
55 to 59	21,795 (11.6)	19,351 (11.3)	2444 (15.0)	< 0.001
60 to 64	30,897 (16.5)	27,218 (15.9)	3679 (22.6)	< 0.001
65 to 69	40,440 (21.5)	36,770 (21.4)	3670 (22.6)	0.001
70 to 74	34,534 (18.4)	32,388 (18.9)	2146 (13.2)	< 0.001
75 to 79	11,627 (6.2)	11,222 (6.5)	405 (2.5)	< 0.001
> = 80	11,681 (6.2)	11,513 (6.7)	168 (1.0)	< 0.001
**Sex**				
Male	173,852 (92.6)	158,585 (92.5)	15,267 (93.8)	< 0.001
**Race**				< 0.001
American Indian or Alaska Native	2671 (1.4)	2373 (1.4)	298 (1.8)	
Asian	831 (0.4)	765 (0.4)	66 (0.4)	
Black or African American	30,396 (16.2)	27,641 (16.1)	2755 (16.9)	
Native Hawaiian or other Pacific Islander	1854 (1.0)	1673 (1.0)	181 (1.1)	
White	142,128 (75.7)	130,255 (75.9)	11,873 (73.0)	
Unknown	9894 (5.3)	8795 (5.1)	1099 (6.8)	
**Hispanic**	7389 (3.9)	6521 (3.8)	868 (5.3)	< 0.001
**Married**	98,866 (52.7)	92,126 (53.7)	6740 (41.4)	< 0.001
**Marginally housed**	10,126 (5.4)	8538 (5.0)	1588 (9.8)	< 0.001
**Pain diagnoses**				
Back pain	123,286 (65.7)	113,185 (66.0)	10,101 (62.1)	< 0.001
Neck pain	38,463 (20.5)	35,071 (20.4)	3392 (20.8)	0.235
Osteoarthritis	53,070 (28.3)	49,312 (28.8)	3758 (23.1)	< 0.001
Neuropathy	37,299 (19.9)	34,813 (20.3)	2486 (15.3)	< 0.001
Headache	27,538 (14.7)	24,488 (14.3)	3050 (18.7)	< 0.001
**Mental health**				
Psychosis	7670 (4.1)	6868 (4.0)	802 (4.9)	< 0.001
Depression	59,786 (31.8)	54,086 (31.5)	5700 (35.0)	< 0.001
Bipolar	6927 (3.7)	6082 (3.5)	845 (5.2)	< 0.001
PTSD	44,259 (23.6)	39,768 (23.2)	4491 (27.6)	< 0.001
Anxiety	32,102 (17.1)	28,926 (16.9)	3176 (19.5)	< 0.001
Self-harm	254 (0.1)	215 (0.1)	39 (0.2)	< 0.001
**Behavioral risk factors**				
Opioid use disorder	11,672 (6.2)	10,296 (6.0)	1376 (8.5)	< 0.001
Benzodiazepine use disorder	970 (0.5)	821 (0.5)	149 (0.9)	< 0.001
Hallucinogen use disorder	52 (0.0)	42 (0.0)	10 (0.1)	0.014
Stimulant use disorder	3328 (1.8)	2785 (1.6)	543 (3.3)	< 0.001
Other drug use disorder	2634 (1.4)	2163 (1.3)	471 (2.9)	< 0.001
Alcohol use disorder or Elevated AUDIT-C^a^	26,020 (13.9)	22,534 (13.1)	3486 (21.4)	< 0.001
Current tobacco use	45,181 (24.1)	37,667 (22.0)	7514 (46.2)	< 0.001
**Substance present in urine drug test- day of index**				
Positive amphetamine lab	2877 (1.5)	2479 (1.4)	398 (2.4)	< 0.001
Positive benzodiazepine lab	8779 (4.7)	7736 (4.5)	1043 (6.4)	< 0.001
Positive cocaine lab	1095 (0.6)	841 (0.5)	254 (1.6)	< 0.001
**Mean daily morphine equivalent dose (SD)**	42.86 (77.78)	42.57 (76.39)	45.92 (91.19)	< 0.001

^a^We defined high risk drinking based on an audit score of 4 or above for women and 5 or above for men or an ICD code related to alcohol use disorder/dependence

To identify substance use disorders, we used the presence of a single ICD code in the past two years. We required only a one code for substance use disorders as these conditions tend to be under-coded by providers. We searched for codes indicating common substance use disorders including opioid, benzodiazepine, hallucinogen, stimulant, and alcohol use disorders (Table 1). The category “other drug use disorders” included any drugs not covered by preceding categories (e.g., inhalant use). Notably, as ICD-9 codes contain outdated language such as substance “abuse,” or “dependence,” we reported these diagnoses as “use disorders” in accordance with current standards of addiction-related terminology [23, 24].

In addition to using ICD codes, we included individuals with elevated scores on the Alcohol Use Disorders Identification Test – Concise (AUDIT-C) in the alcohol use category [25]. Screening using the AUDIT-C is required annually by the VHA, and the tool has been well-validated for the detection of risky drinking among Veterans [26]. We defined elevated scores based on the high-risk AUDIT-C scores of ≥ 4 for women and ≥ 5 for men [27]. We used a previously developed algorithm to identify tobacco use [28]. We used UDT data to identify Veterans who had evidence of other substances (cocaine, amphetamines or benzodiazepines) in their urine on the date of the index urine drug screen. As the presence of a substance on UDT alone does not indicate a use disorder, we did not count lab results in the use disorder categories, but rather reported them separately as “Substance Present in Urine Drug Test- day of index” (Table 1 and Fig. 3).

Veterans were classified as “marginally housed” if an ICD code related to housing insecurity or homelessness was present or if they received housing services. We defined self-harm based on ICD code.

### Statistical analysis

We assessed the annual prevalence of cannabis use among opioid-prescribed Veterans between 2014 to 2018. To estimate annual prevalence, we first identified all Veterans who received a UDT in a given year and had an opioid prescription within 90 days before the UDT (Fig. 1). For each Veteran with more than one UDT in a given year, the first UDT result was used such that each Veteran appears only once each year. We chose the first UDT of each year as Veterans may complete variable numbers of total UDTs per year, and selection of the first data point allowed us to gather a non-biased, standardized data point for each Veteran. Annual prevalence was estimated by identifying the proportion first UDTs positive for cannabis out of all the tests sampled each year (2014 through 2018).

We described the characteristics of the sample stratified by cannabis present in the urine drug test. Characteristics were summarized using frequencies and proportions and compared as a function of cannabis use using chi-square tests for categorical variables and t-test for continuous variables.

We examined the associations between sociodemographic factors, pain diagnoses, mental health diagnoses, and substance use disorders with cannabis in the UDT in 2018 because this was the most recent year of data in the sample at the time of the analyses. We assessed the association between these factors and cannabis in the UDT using multivariable logistic regression. For the multivariable analysis, we combined the different forms of pain into one category (any pain diagnosis). In a secondary analysis, we examined the association of cannabis in the UDT with the factors outlined above stratified by age (< 55 and > 55). We conducted this analysis because less is known about factors associated with cannabis use among older adults. We used RStudio version 1.4.17 for all analyses.

### Institutional approvals

The Institutional Review Board of the University of California, San Francisco approved this study.

## Results

### Geographical variation in prevalence of cannabis use

**Fig. 2:**
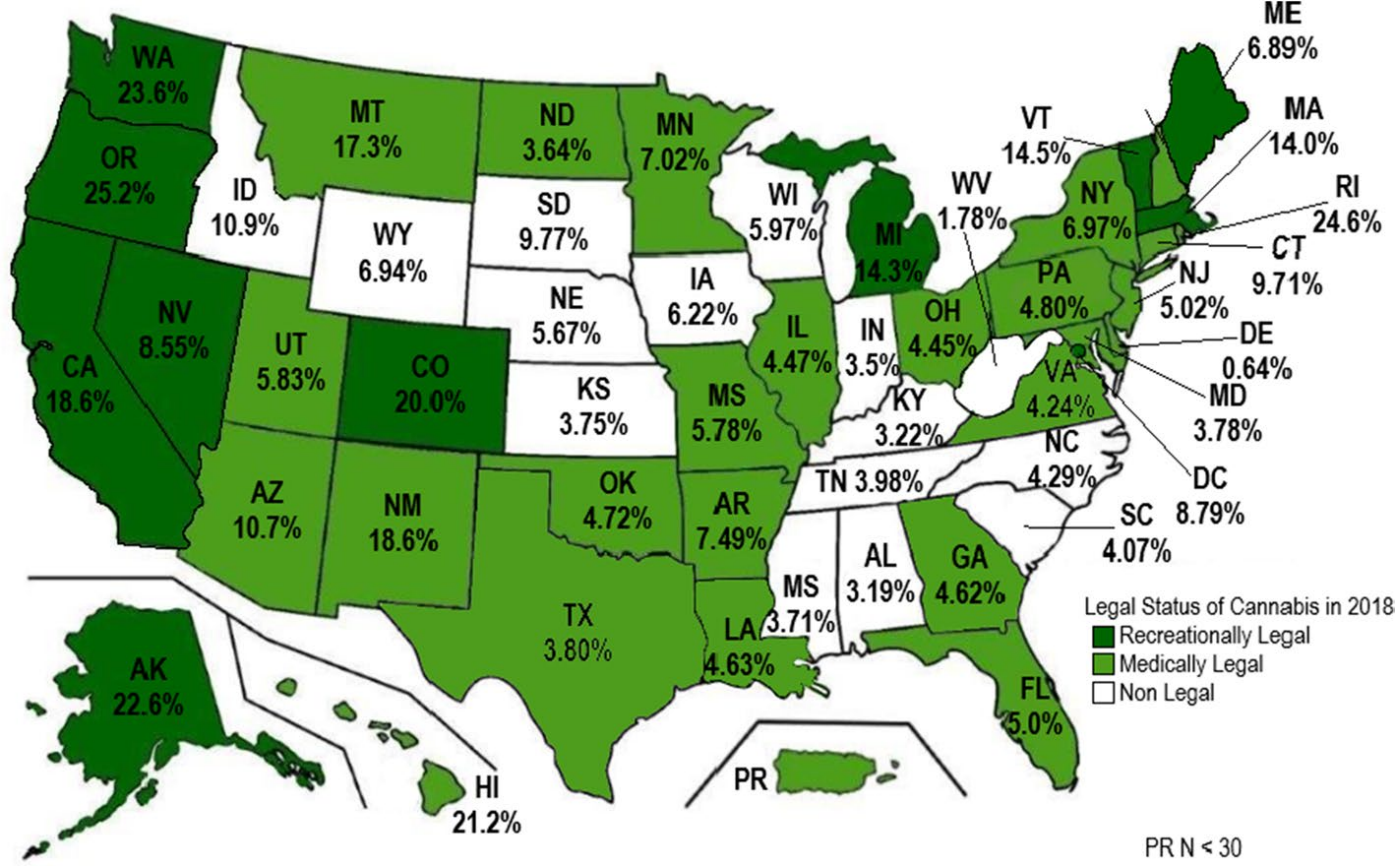
Prevalence of Cannabis use in Veterans 18 years and older on prescription opioid therapy across US States and territories in 2018. Original figure created using microsoft word 2021 and microsoft paint 2021

Among Veterans prescribed at least one opioid, the annual prevalence of cannabis positive UDT was 9.7% (95% CI, 9.55% to 9.78%) in 2014, 8.8% (95% CI, 8.68% to 8.90%) in 2015, 8.6% (95% CI 8.50% to 8.71%) in 2016, 8.5% (95%CI 8.41% to 8.64%) in 2017, and 8.7% (95%CI 8.53% to 8.79%) in 2018 (Fig. 1). In 2018, there was heterogeneity in cannabis prevalence among opioid-prescribed Veterans by state (Fig. 2) with the highest prevalence in Oregon (25.2%), Washington (23.6%) and California (18.6%). In 2018, cannabis was legal for recreational and medical purposes in 10 states and the District of Columbia (D.C.), cannabis was legal for medical purposes in 25 states and Puerto Rico, and cannabis was non-legal in 15 states (Fig. 2). The prevalence of cannabis use among opioid-prescribed Veterans was 18.2% in recreationally legal states, 6.1% in medically legal states, and 4.5% in non-legal states (*p* < 0.001).

### Demographic factors and cannabis use

The mean age of the Veterans with an opioid prescription who completed UDT in 2018 was 63.3 years. The majority were male (92.6%) and white (75.7%) (Table 1).

**Fig. 3:**
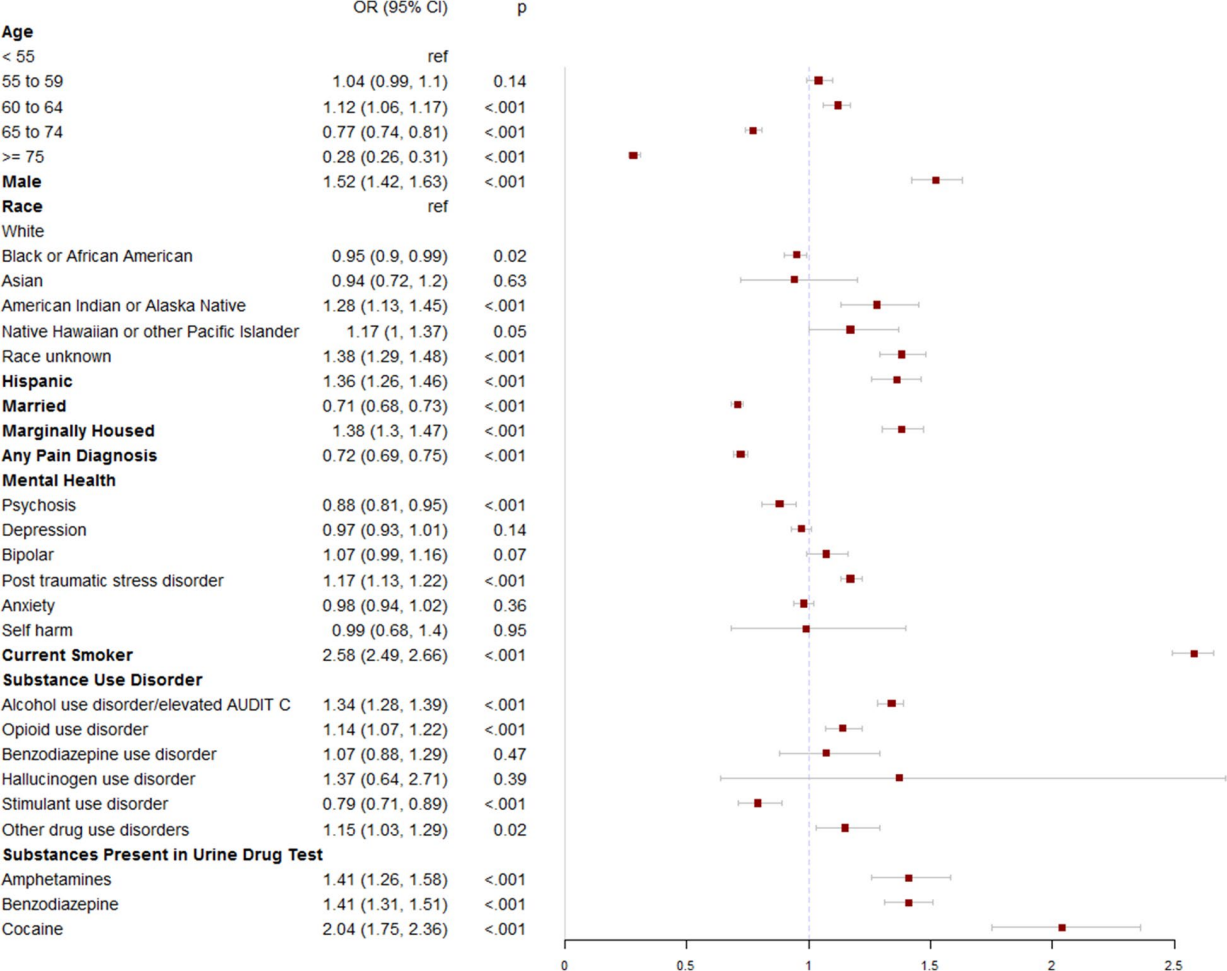
Adjusted associations between sociodemographic, pain, mental health, and behavioral factors and presence of cannabis in the urine drug test. All factors listed were included in the model

Age below 55 years, male gender (adjusted odds ratio [AOR] 1.52; 95% confidence interval [CI] 1.42–1.63, *p* < 0.001), being unmarried (AOR 0.71; 95% CI 0.68–0.73, *p* < 0.001), marginally housed (AOR 1.38; 95% CI 1.3–1.47, *p* < 0.001), of American Indian or Alaska Native race (AOR 1.28; 95% CI 1.13–1.45, *p* < 0.001), and of Hispanic or Latino ethnicity (AOR 1.36; 95% CI 1.26–1.46, *p* < 0.001) were associated with cannabis use (Fig. 3).

### Other substance use disorders and cannabis use

After adjusting for baseline characteristics, patients with opioid use disorder (AOR 1.14; CI 1.07–1.22, *p* < 0.001), alcohol use disorder or elevated AUDIT C (AOR 1.34; 95% CI 1.28–1.39, *p* < 0.001), other drug use disorders (AOR 1.15; 95% CI 1.03–1.29, *p* = 0.02), or who were current smokers (AOR 2.58; 95% CI 2.49–2.66, *p* < 0.001) had higher odds of cannabis positive UDT. Those with UDT positive for amphetamines (AOR 1.41; 95% CI 1.26–1.58), benzodiazepines (AOR 1.41, 95% CI 1.31–1.51), or cocaine (AOR 2.04, 95% CI 1.75–2.36), were more likely to have cannabis positive UDT (Fig. 3).

### Other mental health diagnoses and cannabis use

In adjusted analysis, psychosis, depression, bipolar disorder, anxiety, and self-harm were not associated with increased cannabis positive UDT. Veterans with a post-traumatic stress disorder (PTSD) diagnosis had higher odds of cannabis positive UDT (AOR 1.17; 95% CI 1.13–1.22, *p* < 0.001) (Fig. 3).

### Pain diagnoses, opioid dose, and cannabis use

Among the study population, 84% of Veterans had documented ICD codes for pain diagnoses. In adjusted analysis, presence of any pain diagnosis (back pain, neck pain, osteoarthritis headache or neuropathy) (AOR 0.72; 95% CI 0.69–0.75, *p* < 0.001) was associated with reduced likelihood of having cannabis in the UDT. (Fig. 3). Veterans who used cannabis were prescribed a higher dose of prescription opioids compared to those without (mean daily morphine equivalent dose 45.9 mg versus 42.5 mg; *p* < 0.001).

### Cannabis use stratified by age

We found that the factors associated with cannabis use were similar among veterans younger than 55 years old and those aged 55 and older with one exception. Veterans 60 to 64 were slightly more likely to have cannabis in the UDT compared to those aged < 55 years (OR 1.12, 95% CI 1.06–1.17 (Fig. 3).

## Discussion

This population-based study of biologically verified cannabis use among Veterans prescribed opioids within the VHA demonstrates an overall national prevalence of 8.7% in 2018, similar to non-Veterans with medical conditions (8.8%) [29]. We report higher cannabis use in recreationally legal states compared to non-legal states, with 20–25% of Veterans on opioids using cannabis in California, Oregon, and Washington. Furthermore, our study demonstrated that PTSD, current smoking of tobacco, alcohol, opioid, and other drug use disorders, were associated with cannabis positive UDT.

In contrast to studies of the general adult population [2], we did not find an increase in cannabis use among opioid-prescribed patients over time (between 2014–2018). There are several possible explanations for this finding. The VHA does not mandate the taper of opioids in response to cannabis positive UDT. However, in response to national efforts to improve the safety of patients on opioids [30, 31], some VHA prescribers may taper opioids or counsel against cannabis use if patients use both, contributing to lower prevalence. Additionally, the average age of our study population (63 years) may be older than a non-Veteran general population. However, recent data indicate that cannabis use may be growing more quickly in older persons compared with younger populations [32, 33]. This suggests the added importance of monitoring and addressing the use of cannabis in the aging Veteran population.

Our study indicates an association between PTSD and cannabis use among Veterans prescribed opioids, similar to the association seen in the broader veteran population [34]. This finding suggests the importance of screening and connecting opioid-prescribed Veterans to evidence-based treatments for PTSD, as they may be using cannabis to cope with symptoms [35].

The finding that opioid and alcohol use disorders were associated with cannabis positive UDT suggests that cannabis use may be a marker of elevated risk in this population. Veterans on opioid therapy who use cannabis may benefit from evaluation for opioid use disorder and connection to evidence-based treatments within the VHA [36]. The association with alcohol also raises safety concerns, as both alcohol and cannabis have psychoactive affects and are associated with impaired driving [37]. In combination with alcohol, cannabis has a significantly enhanced impact on cognition, attention and concentration, and can potentiate the effects of impairment [38, 39]. These risks are also compounded by opioids, and patients should be educated regarding risks of combined use. While our finding of other substances on UDT are not of themselves indicative of use disorders, Veterans may benefit from further inquiry into patterns of use and counseling regarding the medical risks of using multiple substances [40].

Cannabis use alone is not indicative of cannabis use disorder (CUD). However, given the high prevalence of use and the association of other drug use with cannabis positive UDT, this population may benefit from screening for CUD. This would be congruent with current VHA guidance which recommend discussion of the health impacts of cannabis and documentation of use within veterans’ electronic health records [41]. However, these guidelines do not include specific recommendations on the screening for cannabis use disorder (CUD). Potential screening methods may include clinical interview regarding ongoing use despite consequences [42], standardized tools such as the Cannabis Use Disorder Identification Test (CUDIT) [43], or use of criteria from the Diagnostic and Statistical Manual of Psychiatry (DSM) 5 [44]. If identified, CUD may be managed via referral to cognitive behavioral therapy and motivational enhancement therapy [45]. Patients seeking cannabis cessation may be educated that off-label medications have shown some benefit in treatment of CUD and cannabis withdrawal, though none are currently approved by the Food and Drug Administration [46, 47].

If screening all opioid prescribed patients is not feasible, consideration may be given to initiation of screening in areas with high prevalence such as in the states that have recreationally legalized cannabis use, or in vulnerable populations such as patients with histories of other addiction, psychotic disorders, and PTSD [48, 49]. As a cross-sectional study, ours did not examine the change in prevalence of cannabis positive UDT in each state before and after legalization, though such future analysis may better elucidate the impact of legalization on prevalence.

Our study indicates that current tobacco use was common and had a strong association with cannabis positive UDT. This indicates the need for screening and treatment for nicotine (tobacco) use disorder, particularly given the potential harmful effects of combined inhaled use of cannabis and tobacco [50, 51], although we were unable to distinguish between routes of cannabis use in this study.

Cannabis is being marketed to consumers as a remedy for opioid use, and some states have legalized the dispensing of cannabis for the treatment for opioid use disorder [52]. Our analyses are cross-sectional, and we cannot infer how cannabis use may impact opioid use. However, it is noteworthy that the average opioid doses were higher among those with cannabis positive UDT, and that patients with opioid use disorder were more likely to have cannabis positive UDT. While we did not measure pain severity in this study, previous studies indicate that this alone is an unlikely explanation for combined cannabis and opioid use [11]. Our findings align with other recent studies questioning the utility of cannabis as an alternative to opioids, or as a treatment for opioid use disorder [53, 54]. For patients who report using cannabis use for pain, given emerging research into the efficacy of certain cannabinoids for pain syndromes, prescribers should discuss the relative risks and benefits of using cannabis in a patient-centered manner [6, 55].

Some limitations of this study deserve comment. This a cross-sectional study with all the limitations thereof. While this is a population-based study, some Veterans on opioids may not have received urine drug screens. However, urine drug testing is now an established feature of practice in the VHA for care of Veterans receiving opioids and > 90% of Veterans receiving long-term opioids are screened [18, 56, 57]. In addition, while routine annual urine drug testing is primarily recommended and monitored for Veterans receiving chronic opioid analgesic therapy in primary care, it is possible that some patients received a urine drug screen for other reasons (e.g., mental status and behavioral changes).” Veterans who use cannabis infrequently may not be captured, and our estimates may be an undercount of actual prevalence. We used a UDT to assess for cannabis use, the current gold standard test for assessing cannabis use. However, these tests do not provide information on routes, frequency or forms of use, and may detect positive tests more reliably for those who use frequently [58]. Additionally, given the potential ramifications of a positive result, particularly in states where cannabis remains illegal, some patients may avoid completing a UDT, although an annual UDT is recommended for opioid continuation and compliance is monitored [59]. Though some patients may have prescriptions for benzodiazepines or stimulants, close monitoring remains important given the risks of drug-drug interactions with opioids and the potential misuse of controlled substance prescriptions [60–62]. We used ICD codes, AUDIT-C scores, and urine drug screens to capture substance use disorders. However, primary care physicians may not routinely code for these conditions, leading to a likely underestimate of substance use in this population. While high AUDIT-C scores indicate risk for alcohol use disorder, they are not diagnostic, supporting the need for additional evaluation.

Cannabis use is common among Veterans who receive prescription opioids and is highly prevalent among Veterans who reside in recreationally legal states. Opioid and alcohol use disorders, as well as tobacco and other drug disorders use are more common among patients who use cannabis. Cannabis use may be a marker of high-risk behavior in this population, which may benefit for screening for other substance use disorders and referral to appropriate treatment.

## Supplementary Information

**Additional file 1.** 

## Data Availability

A de-identified dataset can be made available according to the Veterans Health Administration policies and procedures.
