# Supplementary material for: A national population-based study of cannabis use and correlates among U.S. veterans prescribed opioids in primary care
Source: BMC Psychiatry. 2023 Mar 17;23:177. doi: 10.1186/s12888-023-04648-5 (PMC10021973; doi:10.1186/s12888-023-04648-5)

Supplemental Figure 1. Adjusted Associations Between Sociodemographic, Pain, Mental Health, and Behavioral Factors and Presence of Cannabis in the Urine Drug Screen for Veterans <55 years old. All factors listed were included in the model.


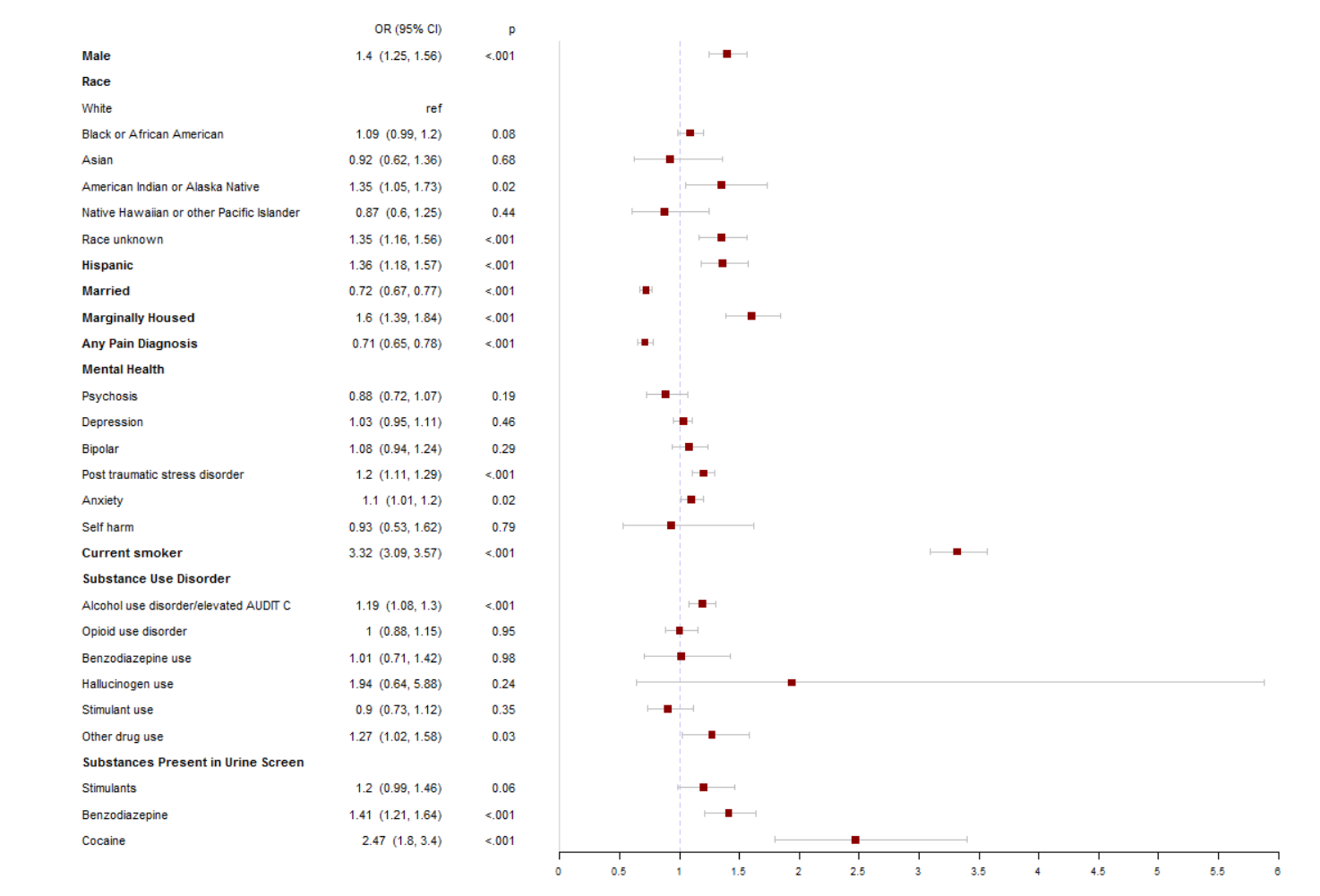


Supplemental Figure 2. Adjusted Associations Between Sociodemographic, Pain, Mental Health, and Behavioral Factors and Presence of Cannabis in the Urine Drug Screen for Veterans 55 years or older. All factors listed were included in the model.


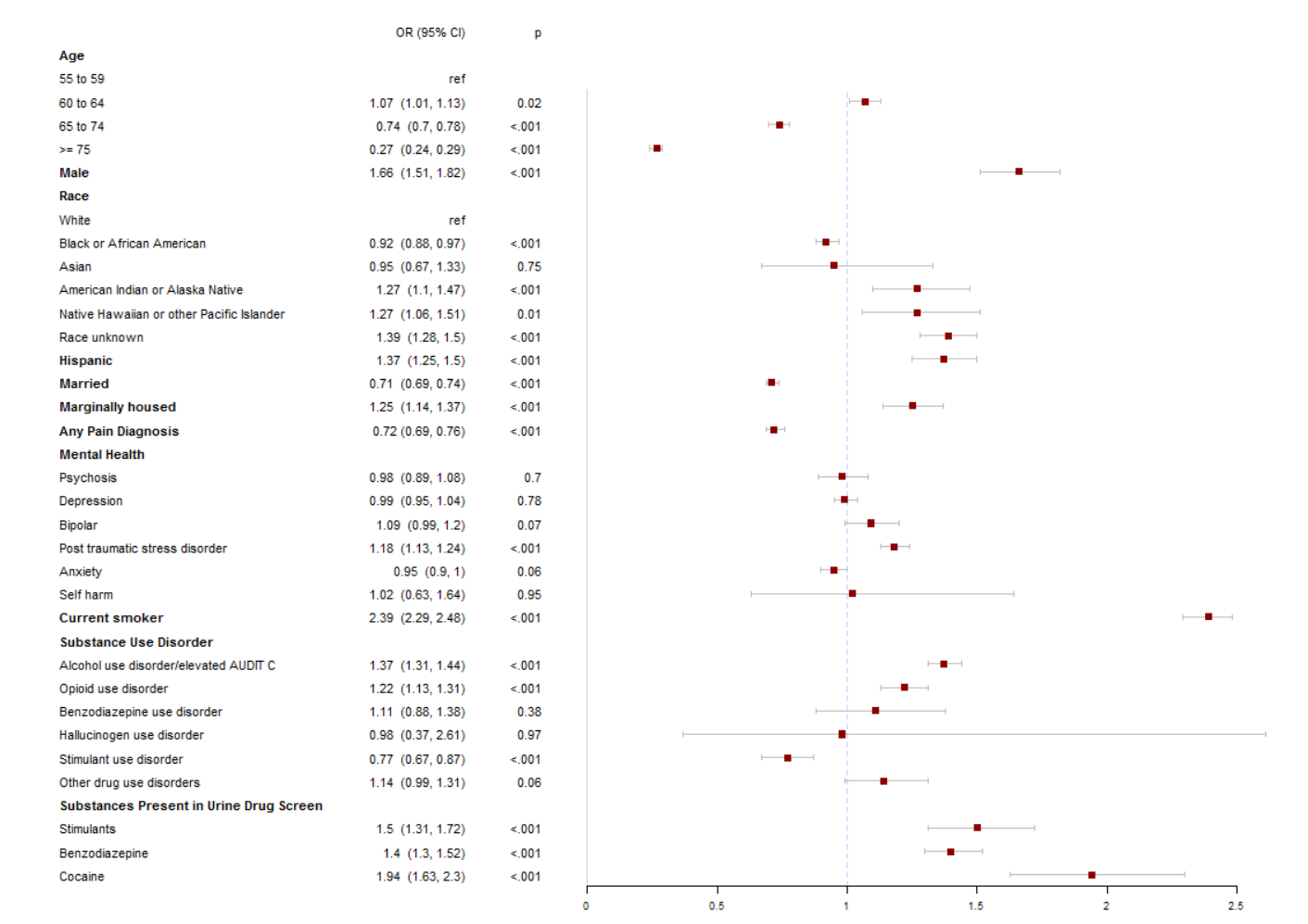

Supplement: Supplementary file 1 — Additional file 1. [file 12888_2023_4648_MOESM1_ESM.docx]
